# Small Molecule Inhibitors of Nicotinamide N-Methyltransferase Enzyme for the Treatment of Osteosarcoma and Merkel Cell Carcinoma: Potential for the Development of a Targeted Therapeutic Strategy

**DOI:** 10.3390/biom15111553

**Published:** 2025-11-05

**Authors:** Veronica Pompei, Monia Cecati, Emma Nicol Serritelli, Eleonora Gerini, Roberto Campagna, Valentina Pozzi, Matthijs J. Van Haren, Nathaniel I. Martin, Monica Emanuelli, Davide Sartini

**Affiliations:** 1 Department of Clinical Sciences, Polytechnic University of Marche, 60126 Ancona, Italy; v.pompei@univpm.it (V.P.); emmaserritelli@gmail.com (E.N.S.); ele.gerini@gmail.com (E.G.); r.campagna@univpm.it (R.C.); m.emanuelli@univpm.it (M.E.); 2 Department of Human Sciences and Promotion of the Quality of Life, San Raffaele Roma Open University, 00166 Rome, Italy; moniacecati@gmail.com; 3 Biological Chemistry Group, Institute of Biology, Leiden University, 2311 Leiden, The Netherlands; m.vanharen@cantonitx.nl (M.J.V.H.); n.i.martin@biology.leidenuniv.nl (N.I.M.); 4 New York-Marche Structural Biology Center (NY-MaSBiC), Polytechnic University of Marche, 60121 Ancona, Italy

**Keywords:** nicotinamide N-methyltransferase, enzyme inhibitors, osteosarcoma, Merkel cell carcinoma, chemosensitivity, targeted therapy

## Abstract

Nicotinamide N-methyltransferase (NNMT) enzyme catalyzes the N-methylation of nicotinamide and its overexpression has been reported in many neoplasms, favoring traits featuring an aggressive tumor cell phenotype. Our recent data demonstrated that NNMT upregulation in osteosarcoma (OS) and Merkel cell carcinoma (MCC) led to a significant increase in cell proliferation and migration ability, together with a reduction in sensitivity to chemotherapeutic treatment. Based on these findings, we investigated the impact of small molecule NNMT inhibitors 5-amino-1-methyl quinolinium (5-AMQ), 6-methoxynicotinamide (6MeONa) and Eli Lilly’s pyrimidine 5-carboxamide (EL-1) on U-2 OS and Saos-2 OS cell lines and MCC13 and MCC26 MCC cell lines. Following incubation of the cells with these compounds, cell viability, reactive oxygen species (ROS) production and apoptosis induction were evaluated. Cells were then subjected to combined treatment with inhibitors and cisplatin (CDDP), and viability and ROS levels were further analyzed. Our results clearly illustrate that cells treated with NNMT inhibitors underwent significant reductions in viability, increased ROS production and activation of apoptotic pathways. Given the association of NNMT with cancer aggressiveness, inhibiting its catalytic activity might present a novel strategy for counteracting cancer growth and chemoresistance, providing the rationale for an effective anti-cancer therapy based on the use of specific NNMT inhibitors.

## 1. Introduction

The enzyme nicotinamide N-methyltransferase (NNMT) catalyzes the reaction of N-methylation of nicotinamide (NA) by using S-adenosyl-L-methionine (SAM) as a methyl donor, thus producing N1-methylnicotinamide (MNA) and S-adenosyl-L-homocysteine (SAH) [1]. In humans, MNA is directly excreted through urine or transformed to the oxidized species N1-methyl-2-pyridone-5-carboxamide or N1-methyl-4-pyridone-3-carboxamide by the action of aldehyde oxidase [2]. Together with MNA, pyridine compounds undergo urinary excretion, thus presenting major NA catabolites [3].

The activity of NNMT substantially influences intracellular NA levels, determining its urinary excretion upon N-methylation. NA is a basic precursor of nicotinamide adenine dinucleotide (NAD^+^). The conversion of NA to MNA prevents its use in NAD^+^ synthesis, making it evident that NNMT activity plays a fundamental role in regulating the equilibrium between biosynthesis and breakdown of this pyridine nucleotide coenzyme, thus indirectly affecting a wide range of intracellular events [4]. Indeed, in addition to serving a pivotal role in redox reactions featuring metabolism [5], NAD^+^ takes also part in other processes, such as histone deacetylation, as a key event in transcriptional regulation of gene expression and poly(ADP-ribose) polymerization in the context of mechanisms of DNA repair in response to structural damage [6].

Cloning, expression and purification of human recombinant NNMT previously led to the resolution of its three-dimensional structure, as a ternary complex bound to both SAH and NA, together with the identification of D197 and Y20 as key amino acid residues involved in the catalysis [7]. Further studies served to elucidate the underlying enzyme kinetics showing that NNMT follows an ordered mechanism with SAM binding to NNMT before NA [8]. Additional work within our group and by others has led to optimized assays for NNMT along with the development of a variety of NNMT inhibitors whose structure, at least in part, is based on that of the reaction substrates or products [9,10,11].

Among human diseases, NNMT was found to be upregulated in many solid tumors, such as glioblastoma [12], papillary thyroid cancer [13], gastric cancer [14], colorectal cancer [15] and prostate cancer [16]. Experimental studies carried out to investigate the significance of such enzyme overexpression, as well as the impact of NNMT dysregulation on tumor cell phenotype, clearly demonstrated that NNMT positively influences fundamental events promoting and sustaining carcinogenesis, such as cell proliferation, migration, invasiveness, apoptosis inhibition and cell cycle progression, together with chemo and radioresistance [17,18,19,20,21,22,23], strongly supporting the hypothesis that the enzyme may have great potential as a molecular target for effective anticancer treatment.

Results reported in our recent published works showed high NNMT expression levels in association with Merkel cell carcinoma (MCC) and osteosarcoma (OS). Subsequent studies performed with MCC and OS cell lines demonstrated that the induction of shRNA-mediated NNMT silencing decreased cell proliferation, viability and migration, accompanied by enhanced cell sensitivity to treatment with chemotherapy drug cisplatin (CDDP) [24,25]. The aim of the work reported in the present study was to evaluate the effect induced by known NNMT inhibitors on MCC and OS. To this end, MCC (MCC13 and MCC26) and OS (U-2 OS and Saos-2) cell lines were treated with 5-amino-1-methyl quinolinium (5-AMQ), 6-methoxynicotinamide (6MeONa) and Eli Lilly’s pyrimidine 5-carboxamide (EL-1) and effects on cell viability, apoptosis activation, oxidative stress and resistance to chemotherapeutic treatment (CDDP) were evaluated.

## 2. Materials and Methods

### 2.1. Cell Cultures and Inhibitors

Human OS cell lines, U-2 OS and Saos-2, were purchased from American Type Culture Collection (Manassas, VA, USA), and human MCC cell lines, MCC13 and MCC26, were provided by Prof. Baki Akgül, Institute of Virology, University of Cologne, Cologne, Germany. Cell lines were cultured in D-MEM medium supplemented with 10% fetal bovine serum, L-glutamine 2 mM and gentamicin 50 µg/mL. All cell lines were maintained at 37 °C in a humified atmosphere with 5% of CO_2_.

5-AMQ, 6MeONa and EL-1 compounds, synthesized as previously reported, were dissolved in dimethyl sulfoxide (DMSO) and used to preliminarily treat MCC and OS cells, based on their in vitro IC50 (1.2 μM for 5-AMQ, 2.5 μM for 6MeONa and 74 nM for EL-1) [26,27,28]. Concentration values ranging between 1 μM and 1 mM were explored through MTT assay. Treatment with 1 μM concentration of all compounds did not alter metabolic activity of cancer cells within the explored time course (0–72 h). The final concentration values selected for cell-based assays were 10 and 100 μM. Small molecule NNMT inhibitors were therefore dissolved in DMSO at 100 mM concentration. This stock solution was then diluted in culture medium to final concentration values of 10 and 100 μM. For each sample, DMSO was kept constant at 0.1% final concentration. The effect of small molecules on cell viability, apoptosis activation, oxidative stress and resistance to chemotherapeutic treatment was tested by comparing inhibitor-treated cells with cells grown in regular medium containing 0.1% DMSO, which was considered as controls.

### 2.2. MTT Assay

Cell viability was analyzed by using the (4,5-dimethylthiazol-2-yl)-2,5-diphenyl tetrazolium bromide (MTT) assay. A total of 3 × 10^3^ cells/well were seeded in 96 well plates and, the day after seeding, cells were treated with DMSO 0.1% (controls), or with 5-AMQ, 6-MeONa and EL-1 (10 and 100 μM). Then, the conversion of MTT to formazan crystals was measured (0 h time point). After discarding the medium, 100 μL of D-MEM with 1:12 MTT reagent (5 mg/mL in phosphate buffer saline) was added to each well. Following 2 h of incubation at 37 °C, medium was replaced with isopropanol to dissolve formazan crystals. Absorbance was assessed at 540 nm in a plate reader. Subsequently, the analysis was performed 24, 48 and 72 h after time 0. Results were expressed as percentage with respect to the controls. The absorbance value of each sample at 0 h corresponds to 100% and represents the control. Each experiment was carried out in triplicate and, independently, repeated three times.

### 2.3. Monolayer Wound Healing Assay

In order to verify whether treatment with small molecule NNMT inhibitors could influence cell migration capability, cell lines were seeded into 6-well plates (1.5 × 10^5^ cells/well for U-2 OS, MCC13 and MCC26, and 3 × 10^5^ cells/well for Saos-2). The day after, cells started to be treated with 100 μM 5-AMQ, 6-MeONa or EL-1 for 72 h. After treatment completion, medium was removed and replaced with new media, and cells were allowed to attach until 100% confluency. Cell monolayers were then scratched using sterile 200 μL pipette tip to create a vertical wound. After scratching, wounded monolayers were washed three times and incubated in D-MEM and 0.5% FBS. Wounded monolayers were photographed at 0, 8, 24, 32 and 48 h after scratching. Images were analyzed using ImageJ software v1.54 (Rasband, W.S., ImageJ, U.S. National Institutes of Health, Bethesda, MD, USA, https://ij.imjoy.io/, accessed on 20 October 2025), and each experiment was performed in triplicate and independently repeated three times.

### 2.4. Quantitative Measurements of MNA Levels in Cultured Cells

A total of 10^5^ cells of the different cell lines were seeded in each well of a 6-well plate and were incubated at 37° C in an incubator for 24 h. The next day, cells were subjected to treatment with 0,1% DMSO, or with 5-AMQ, 6-MeONa and EL-1 (100 μM). After 72 h of treatment, cells in each well were detached with trypsin, counted through the Burker’s chamber and Trypan blue, and centrifuged at 1000× *g* for 3 min at 4 °C. After discarding supernatants, cell pellets were stored at −80 °C until use. Cellular MNA levels were further evaluated by using ultra-high performance–hydrophilic interaction liquid chromatography–tandem mass spectrometry technique, as previously described [10]. Each experiment was carried out in triplicate and, independently, repeated three times.

### 2.5. Apoptosis Assay

Apoptotic activation in cell populations treated with DMSO or NNMT inhibitors was determined by using the Annexin V-FITC Early Apoptosis Detection Kit (Cell Signaling, Danvers, MA, USA). After 72 h of treatment with NNMT inhibitors (100 μM) or DMSO 0.1%, cells were harvested by using trypsin, counted through the Burker’s chamber and Trypan blue and then collected by centrifugation. According to the manufacturer’s instructions, after a wash in cold PBS 1×, cell pellets were resuspended in 1× Annexin V Binding Buffer (Cell Signaling, Danvers, MA, USA) at the concentration of 10^5^ cells/mL. Subsequently, 12.5 μL of Propidium Iodide (PI) Solution and 1 μL of Annexin V-FITC Conjugate were added to 96 μL of each cell suspension and then incubated for 10 min on ice in the dark. Each sample was diluted at the final volume of 250 μL with cold 1× Annexin V Binding Buffer and immediately analyzed with flow cytometry by using Guava easyCyte (Millipore Merk Life Science S.r.l., Milan, Italy). Each experiment was carried out in triplicate and, independently, repeated three times.

### 2.6. Expression Levels of Caspases Through Real-Time PCR and Western Blot Analysis

Total RNA from cell pellets (1 × 10^6^) was isolated using SV Total RNA Isolation System (Promega, Madison, WI, USA) according to the manufacturer’s instructions. Quality and quantity of RNA were evaluated through spectrophotometer at 230, 260 and 280 nm. cDNA was obtained through reverse transcription of 2 µg of total RNA with M-MLV Reverse transcriptase (Promega, Madison, WI, USA) and random primers, for 60 min at 37 °C.

Subsequently, a Real-Time PCR assay, carried out using the CFX96 Real-Time PCR Detection System (Bio-Rad, Hercules, CA, USA), was performed to quantitatively evaluate caspase-3, caspase-8 and caspase-9 expression. Each sample was run in duplicate using SsoFast EvaGreen Supermix (Bio-Rad Laboratories, Hercules, CA, USA) following an amplification protocol consisting of 40 cycles at 95 °C for 30 s and 58 °C for 30 s. β-actin was used as reference gene. The sequences of the sense and antisense primers used were as follows: caspase-3 (forward) 5′-TGGAACCAAAGATCATACATGG-3′ and (reverse) 5′-CAGACCGAGATGTCATTCCA-3′, caspase-8 (forward) 5′-GATGATGACATGAACCTGCTG-3′ and (reverse) 5′-TTTGCTGAATTCTTCATAGTCGTT-3′, caspase-9 (forward) 5′-TACTTTCCCAGGTTTTGTTTCC-3′ and (reverse) 5′-AAAGCAACCAGGCATCTGTT-3′, β-actin (forward) 5′-TCCTTCCTGGGCATGGAGT-3′ and (reverse) 5′-AGCACTGTGTTGGCGTACAG-3′. Relative changes in expression levels of caspases were calculated by 2^−Δ(ΔCt)^, where ΔCt = Ct (caspase-3, -8 or -9)—Ct (β-actin) and Δ(ΔCt) = ΔCt (5-AMQ, 6 MeONa or EL-1 treated cells)—ΔCt (0.1% DMSO treated cells). Each experiment was carried out in triplicate and, independently, repeated three times.

In order to evaluate caspase-3 expression and cleavage status, as a definitive marker of apoptosis activation, a Western blot analysis was set up. Cell pellets (2 × 10^6^ cells) were suspended in 200 μL lysis buffer and homogenized using a syringe. Lysates were centrifuged at 16,000× *g* at 4 °C for 10 min, obtaining protein extract. A total of 50 μg samples were subjected to 15% SDS-PAGE and transferred to polyvinylidene fluoride membranes (PVDF). After blocking and washing procedures, blots were probed with rabbit polyclonal antibody against caspase-3 (Cell Signaling Technology) (1:1000 dilution) or with rabbit polyclonal antibody against β-actin (Merck, Milan, Italy) (1:200) overnight at 4 °C, followed by incubation with horseradish peroxidase (HRP)-conjugated goat anti-rabbit IgG (Sigma-Aldrich Merk Life Science S.r.l., Milan, Italy) (1:150,000 dilution) for 2 h. Capspase-3 and β-actin signals were obtained by using SuperSignal West Femto Maximum Sensitivity Substrate (Thermo Fisher Scientific, Waltham, MA, USA). Chemiluminescent bands were then acquired using a ChemiDoc XRS+ System (Bio-Rad Laboratories, Hercules, CA, USA), and signal intensity was quantified using Image Lab Software v6.1.0 (Bio-Rad Laboratories, Hercules, CA, USA). For each sample, caspase-3 expression was calculated as the ratio between the signal intensity of the target protein (caspase-3) and that of the housekeeping protein (β-actin).

### 2.7. Intracellular ROS Production

Intracellular ROS levels were measured by using 2′,7′-dichlorodihydrofluorescein diacetate (DCFH2-DA) (Sigma-Aldrich Merk Life Science S.r.l., Milan, Italy). The day after seeding 3 × 10^3^ cells/well on black 96-well with clear bottom plates (Greiner, Kremsmünster, Austria), cells were subjected to treatment with 0.1% DMSO or specific inhibitors (100 μM) and CDDP (1 and 10 nM), used alone or in combination, followed by an incubation for 72 h. After medium removal, cells were incubated with DCFH2-DA (10 μM) for 45 min in the dark. Then, 100 μL of 1× PBS was added to each well after two washes with the same buffer. The fluorescence at λ_ex_/λ_em_ (485/528 nm) was measured on a plate reader. Each experiment was carried out in triplicate and, independently, repeated three times.

### 2.8. Sensitivity to Chemotherapeutic Treatment

Each cell line was subjected to treatment with CDDP (1 nM and 10 nM) and 100 μM NNMT inhibitors, used alone or in combination, and cell viability was assessed by using MTT assay, at 0 h, 24 h, 48 h and 72 h timepoints. Each experiment was carried out in triplicate and, independently, repeated three times.

### 2.9. Statistical Analyses

All statistical analyses were performed by using GraphPad Prism software version 8.00 for Windows (GraphPad Software, San Diego, CA, USA). Values were expressed as mean ± standard deviation. Differences between examined groups were determined using the one-way analysis of variance (ANOVA). A *p*-value < 0.05 was considered as statistically significant.

## 3. Results

### 3.1. Effect of NNMT Inhibitors on Cell Viability and Migration Capacity

The phenotypic impact of the small molecule NNMT inhibitors on cancer cells was established by assessing the viability of OS and MCC cell lines by means of the MTT assay at different timepoints (0 h, 24 h, 48 h and 72 h) following treatment with the NNMT inhibitors.

**Figure 1:**
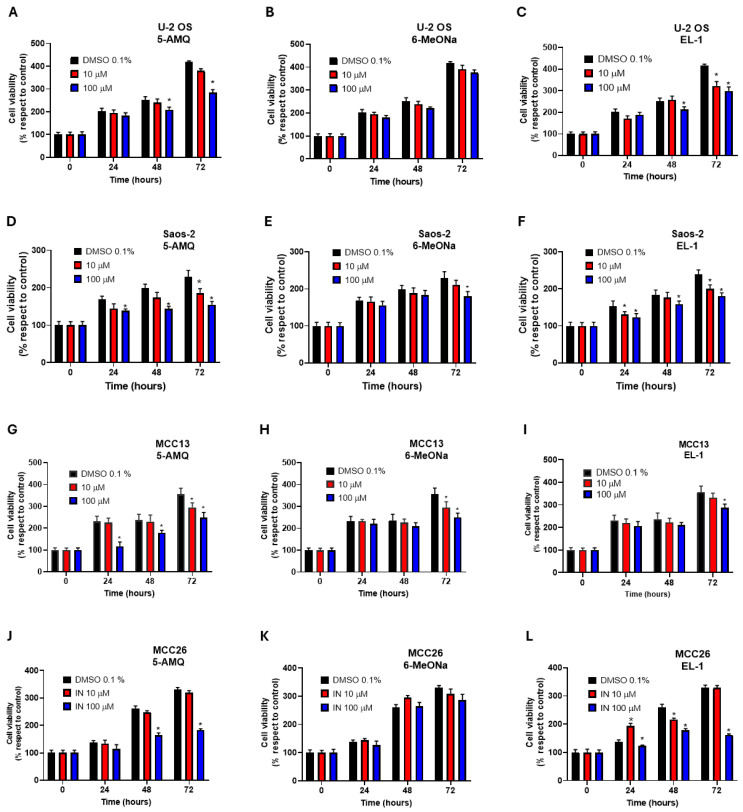
Effect of NNMT inhibitors on viability of OS and MCC cell lines. U-2 OS (**A**–**C**), Saos-2 (**D**–**F**), MCC13 (**G**–**I**) and MCC26 (**J**–**L**) cells were incubated with different concentrations of 5-AMQ, 6-MeONa or EL-1 NNMT inhibitors (10 μM to 100 μM), while control cells were treated with DMSO (0.1%). Cell viability was monitored at 24, 48 and 72 h through MTT assay. All values are expressed as mean ± standard deviation (* *p* < 0.05).

Results obtained from treatment with 100 μM 5-AMQ revealed a significant (*p* < 0.05) reduction in cell viability at all time points for MCC13, while similar effects for the other lines were only observed at 48 h and 72 h. When the compound was used at a lower concentration (10 μM), an effect in Saos-2 and MCC13 cells was only observable at 72 h (Figure 1A,D,G,J). In contrast, the cancer cell lines were found to be largely refractory to treatment with 6MeONa, which led to a slight but significant (*p* < 0.05) reduction in cell viability in Saos-2 (72 h) and MCC13 (48 h and 72 h) (Figure 1B,E,H,K). The third NNMT inhibitor EL-1 was evaluated at 100 μM, resulting in a significant (*p* < 0.05) impact on cell viability at all time points for Saos-2 and MCC26, at 48 h and 72 h for U-2 OS, and at 72 h only for MCC13 (Figure 1C,F,I,L).

**Figure 2:**
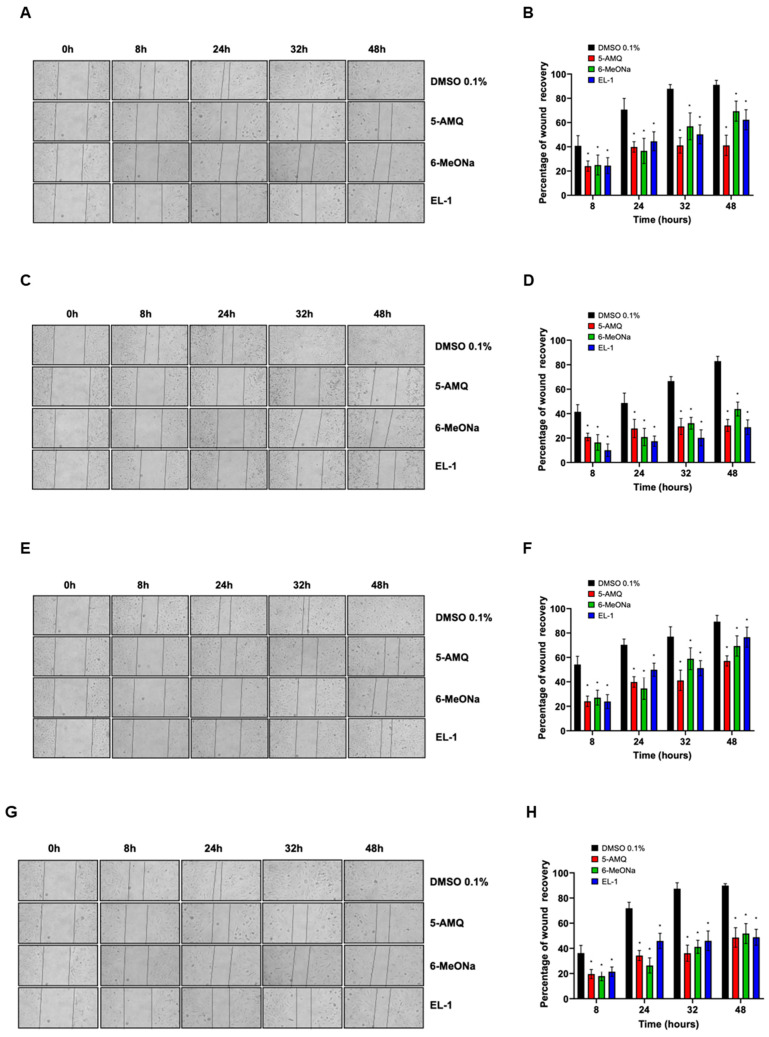
Effect of treatment with small molecule NNMT inhibitors on cell migration. OS and MCC cell lines were subjected to wound healing assay to evaluate their migration potential. After treatment with 100 μM 5-AMQ, 6-MeONa or EL-1 for 72 h, U-2 OS (**A**), Saos-2 (**C**), MCC-13 (**E**) and MCC26 (**G**) cells were photographed immediately after scratch (0 h) and at different time points, ranging between 8 and 48 h. Migration ability was evaluated by measuring percentage of wound recovery compared with 0 h, as reported in bar diagrams ((**B**) for U-2 OS, (**D**) for Saos-2, (**F**) for MCC-13 and (**H**) for MCC-26). Each experiment, in triplicate, was repeated three times. Values are expressed as mean ± standard deviation (* *p* < 0.05).

The migration capability of U-2 OS, Saos-2, MCC-13 and MCC-26 cells was monitored by monolayer wound healing assay. For each sample, data were reported as percentage of wound recovery with respect to 0 h. Compared with control samples (DMSO 0.1%), the migration rate of all cell lines treated with 100 μM 5-AMQ, 6-MeONa or EL-1 was significantly (*p* < 0.05) lower at all tested time points (Figure 2).

### 3.2. Intracellular MNA Levels Following Treatment with Small Molecule Compounds

**Figure 3:**
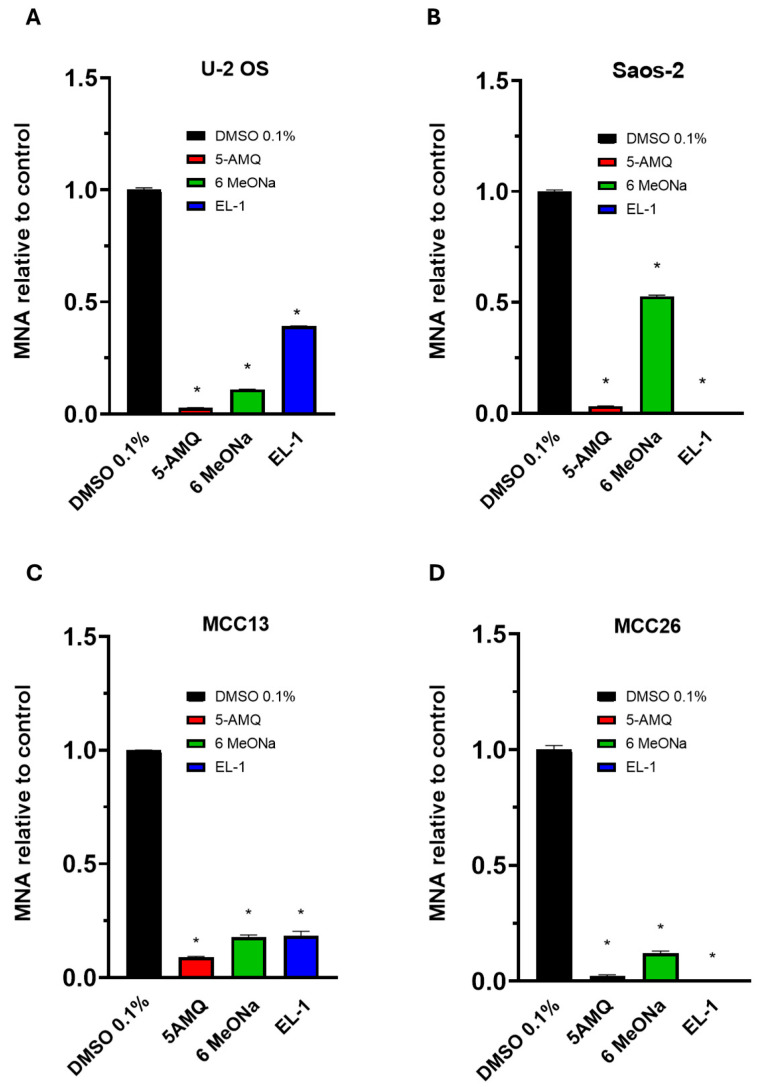
Intracellular MNA content in OS and MCC cells. U-2 OS (**A**), Saos-2 (**B**), MCC13 (**C**) and MCC26 (**D**) cells were incubated with NNMT inhibitors 5-AMQ, 6-MeONa or EL-1 at fixed concentration (100 μM) while control cells were treated with DMSO (0.1%). MNA levels were determined at 72 h after treatment using UHP-HILIC-MS/MS. All values are expressed as mean ± standard deviation (* *p* < 0.05).

We next investigated the effect of 5-AMQ, 6MeONa and EL-1 on cellular NNMT activity by assessing impact on MNA production in all cell lines. Cells were treated with 100 μM of each compound, and MNA levels were determined 72 h after starting treatment. As illustrated in Figure 3, all NNMT inhibitors were able to induce a significant (*p* < 0.05) decrease (at least ≥50%) in the levels of MNA compared to controls for all tumor cells. Interestingly, 5-AMQ exhibited a higher impact in reducing MNA production than detected for 6MeONa. As for EL-1, its inhibitor effect was comparable to or lower than that of 6MeONa for U-2 OS and MCC13, while it led to an abolishment of MNA production in Saos-2 and MCC26.

### 3.3. Apoptosis Activation Induced by Treatment with NNMT Inhibitors

**Figure 4:**
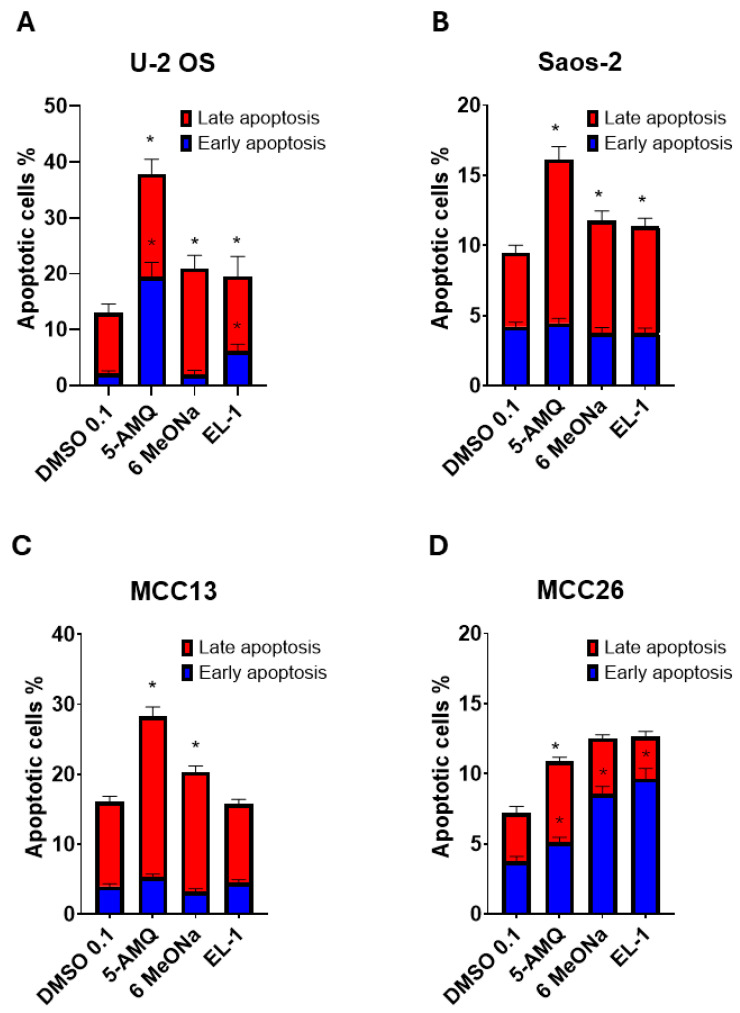
Effect of NNMT inhibitors on apoptosis rate of OS and MCC cell lines. U-2 OS (**A**), Saos-2 (**B**), MCC13 (**C**) and MCC26 (**D**) cells were incubated with NNMT inhibitors 5-AMQ, 6-MeONa or EL-1 at fixed concentration (100 μM) while control cells were treated with DMSO (0.1%). For each sample, the percentage of early (blue) and late (red) apoptotic cells was calculated at 72 h after treatment by using flow cytometry (Annexin V-FITC and Propidium Iodide). All values are expressed as mean ± standard deviation (* *p* < 0.05).

The impact of the three NNMT inhibitors on the rate of apoptotic pathway induction was investigated by treating cells with Annexin V-FITC and PI, followed by flow cytometry. The data reported clearly illustrated that none of the compounds were able to modulate early apoptosis in both Saos-2 (Figure 4B) and MCC13 (Figure 4C). On the contrary, treatment with all three NNMT inhibitors was associated with a significant (*p* < 0.05) increase in early apoptosis in MCC26 (Figure 4D), while in U-2 OS, both 5-AMQ and EL-1 but not 6MeONa, were able to promote an early apoptotic rate (Figure 4A). Concerning late apoptosis, we observed a significant (*p* < 0.05) rate increase following treatment with all compounds for U-2 OS (Figure 4A) and Saos-2 (Figure 4B). Interestingly, in both MCC cell lines, 5-AMQ only led to a marked activation of late apoptosis (Figure 4C,D).

### 3.4. Effect of Treatment with NNMT Inhibitors on Expression Levels of Apoptotic Caspases

**Figure 5:**
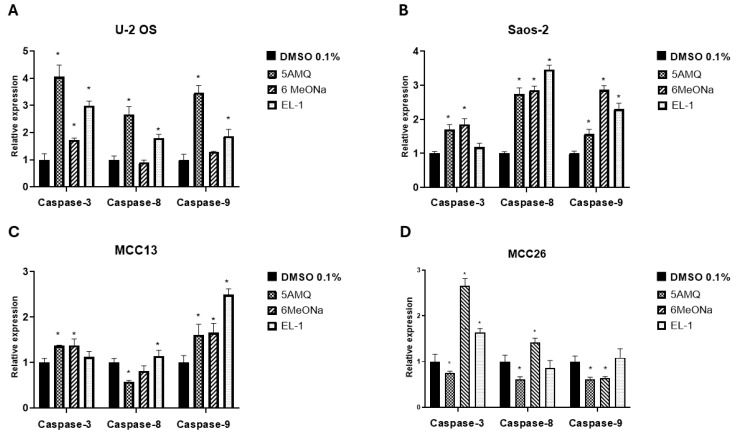
Effect of NNMT inhibitors on caspase activation of OS and MCC cell lines. U-2 OS (**A**), Saos-2 (**B**), MCC13 (**C**) and MCC26 (**D**) cells were incubated with NNMT inhibitors 5-AMQ, 6-MeONa or EL-1 at fixed concentration (100 μM) while control cells were treated with DMSO (0.1%). Expression levels of initiator (caspase-8 and -9) and executioner caspases (caspase-3) were analyzed at 72 h after treatment through Real-Time PCR. All values are expressed as mean ± standard deviation (* *p* < 0.05).

For both OS cell lines, data obtained from Real-Time PCR analyses demonstrated that all three NNMT inhibitors significantly (*p* < 0.05) promoted the expression levels of initiator caspase-3 in U-2 OS (Figure 5A) and executioner caspase-8 and -9 in Saos-2 (Figure 5B). In addition, 6MeONa, but not 5-AMQ and EL-1, did not exert any effect on expression of caspase-8 and -9 in U-2 OS (Figure 5A). In Saos-2, no impact on the transcriptional activation of caspase-3 was induced by EL-1 treatment (Figure 5B). As for MCC13 cells, 5-AMQ and 6MeONa treatment were associated with increased expression of caspase-3 and -9, while EL-1 displayed a significant (*p* < 0.05) effect in enhancing levels of caspase-8 and -9 (Figure 5C). In MCC26, the positive impact on caspase expression exerted by the treatment with NNMT inhibitors seemed to be lower than that detected in other cell lines, with 6MeONa being able to promote caspase-3 and -8 levels, together with the effect induced by EL-1 on the expression of caspase-3 (Figure 5D).

**Figure 6:**
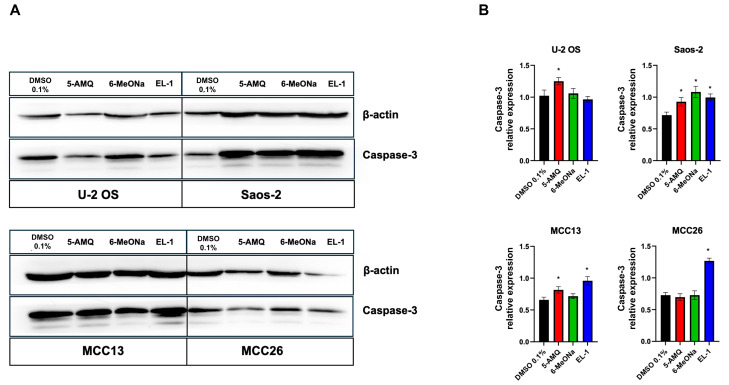
Evaluation of caspase-3 expression in OS and MCC cells upon treatment with NNMT inhibitors**.** Aliquots (50 μg) of protein extract, obtained from U-2 OS, Saos-2, MCC-13 and MCC26 cells treated or not with 100 μM 5-AMQ, 6-MeONa or EL-1, were subjected to 12.5% SDS-PAGE and transferred to PVDF membranes. Blots were probed with rabbit anti-caspase-3 or anti-β-actin antibodies and analyzed with chemiluminescence (**A**). Densitometry was subsequently used to evaluate signal intensity of chemiluminescent bands. (**B**) Caspase-3 expression, calculated as described under Section 2, is expressed as mean ± standard deviation (* *p* < 0.05). Original Western blot images are provided in the Supplementary Materials.

Western blot analyses, coupled with densitometry of immunoreactive bands, demonstrated markedly increased caspase-3 protein expression in U-2 OS treated with 5-AMQ, while MCC26 exhibited a significantly (*p* < 0.05) higher level of caspase-3 following treatment with EL-1. Caspase-3 was overexpressed in Saos-2 cells treated with all tested inhibitors with respect to controls. As for MCC-13, treatment with 6-MeONa only did not lead to an enhancement in caspase-3 expression (Figure 6). However, the band related to cleaved caspase-3 was not detectable.

### 3.5. Modulation of Intracellular Oxidative Stress upon NNMT Inhibitor Treatment

**Figure 7:**
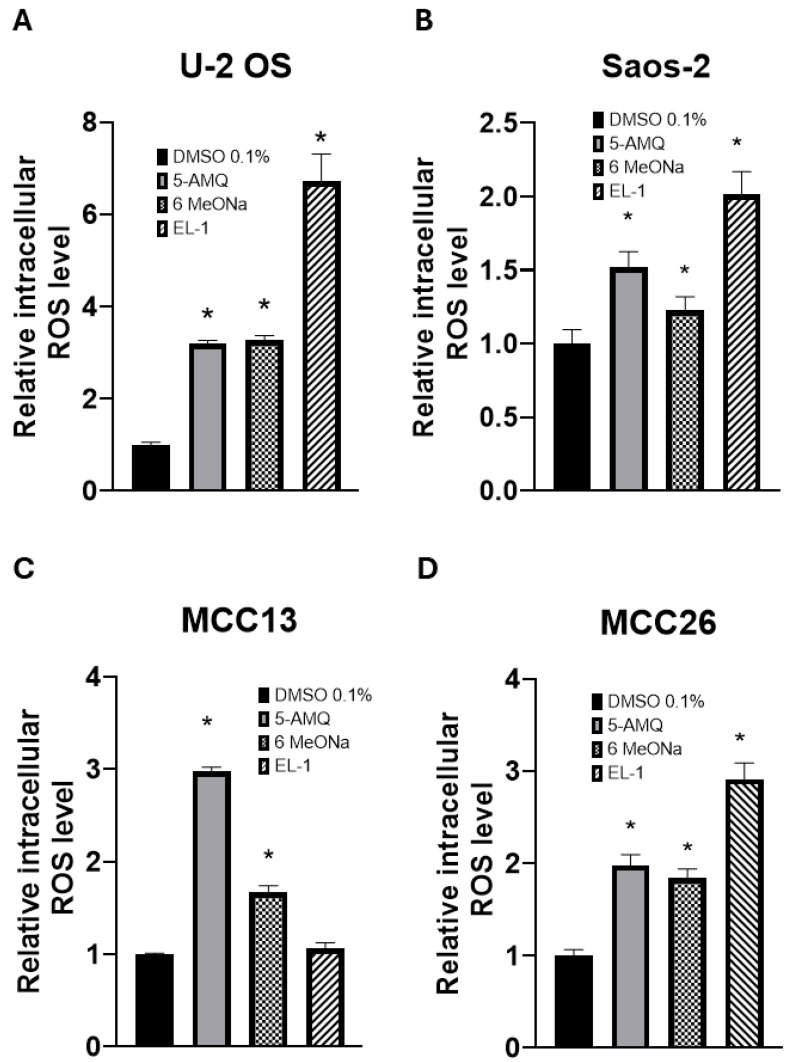
Effect of NNMT inhibitors on oxidative stress of OS and MCC cell lines. U-2 OS (**A**), Saos-2 (**B**), MCC13 (**C**) and MCC26 (**D**) cells were incubated with NNMT inhibitors 5-AMQ, 6-MeONa or EL-1 at fixed concentration (100 μM) while control cells were treated with DMSO (0.1%). ROS production was determined at 72 h after treatment by using DCFH2-DA probe. All values are expressed as mean ± standard deviation (* *p* < 0.05).

The probe DCFH2-DA was used to determine intracellular ROS levels in OS and MCC cell lines treated with NNMT inhibitors. The data reported in Figure 7 clearly demonstrates that both 5-AMQ and 6MeONa were able to significantly (*p* < 0.05) increase ROS production in all cancer cell lines, while EL-1 exerted the same effect in both OS cell lines and in MCC26, but not in MCC13.

### 3.6. Effect of Treatment with NNMT Inhibitors on Chemosensitivity

**Figure 8:**
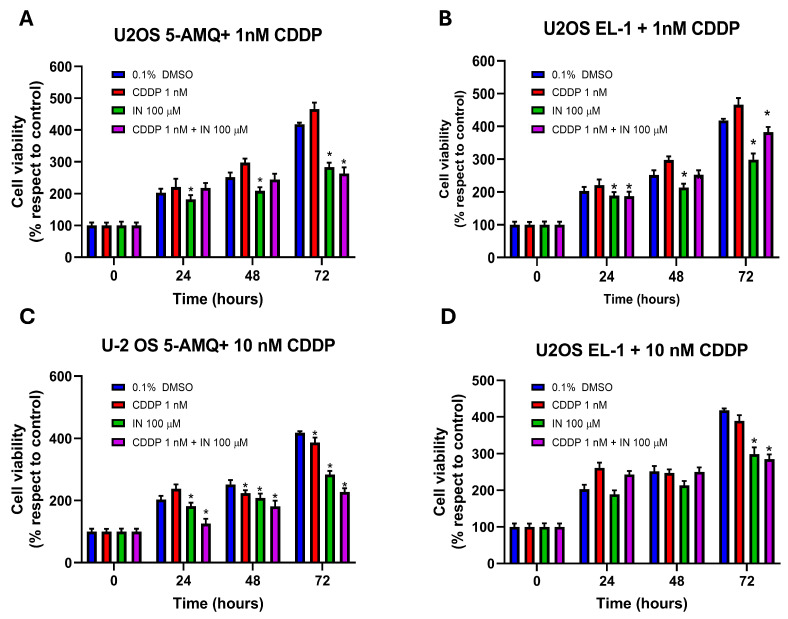

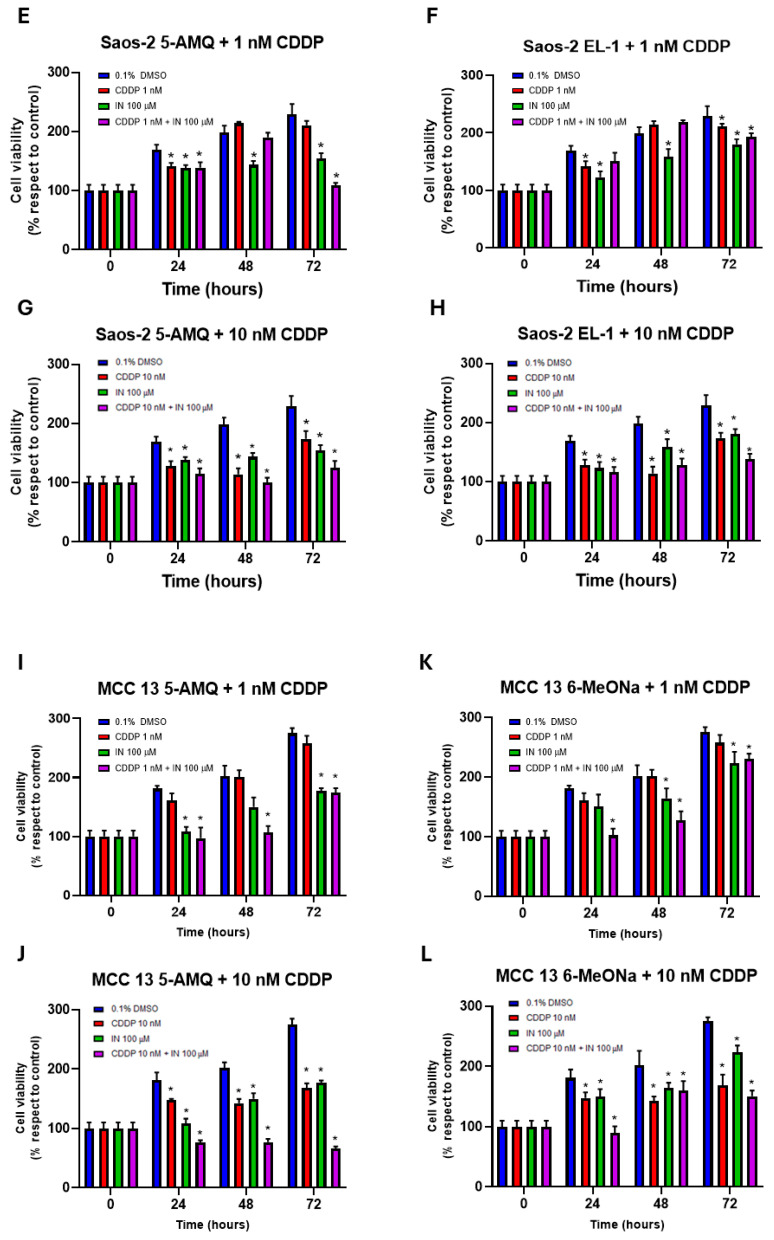

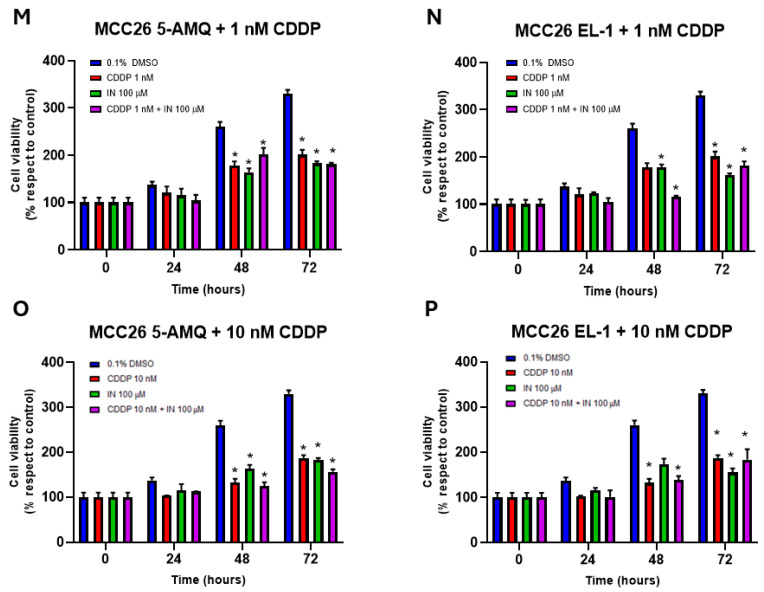
Combined effect of NNMT inhibitors and chemotherapy drug cisplatin (CDDP) on viability of OS and MCC cell lines. U-2 OS (**A**–**D**), Saos-2 (**E**–**H**), MCC13 (**I**–**L**) and MCC26 (**M**–**P**) cells were incubated with NNMT inhibitors 5-AMQ, 6-MeONa or EL-1 at (100 μM) or CDDP (1 nM and 10 nM), used alone or in combination, while control cells were treated with DMSO (0.1%). Cell viability was monitored at 72 h treatment through MTT assay. All values are expressed as mean ± standard deviation (* *p* < 0.05).

To investigate the impact of administration of the NNMT inhibitors on sensitivity to chemotherapy, OS and MCC cell lines were subjected to single and combined treatment with CDDP (1 nM and 10 nM) and 100 µM of each compound and further subjected to MTT assay in order to evaluate metabolic activity of cancer cells at 24 h, 48 h and 72 h after treatment. Regarding U-2 OS, both 5-AMQ and EL-1 were able to significantly (*p* < 0.05) enhance 1 nM CDDP cytotoxicity at all time points (Figure 8A,B), while 5-AMQ only potentiated the antiblastic effect of 10 nM CDDP (Figure 8C). On the contrary, at this CDDP concentration, EL-1 led to a detectable increase in chemosensitivity at 72 h only (Figure 8D). Interestingly, combined treatment with 10 nM CDDP and 100 µM 5-AMQ was associated with a significantly (*p* < 0.05) higher cytotoxic effect than that induced by each single administration (Figure 8C). In Saos-2 cells, the treatment with 100 µM 5-AMQ and EL-1 significantly (*p* < 0.05) increased sensitivity to 1 nM CDDP at all time points (Figure 8E,F). This behavior was kept when treating cells with higher CDDP concentration (10 nM), except for 5-AMQ at 72 h (Figure 8G,H). Combined treatment was able to induce an increased response compared with that generated by a single treatment at 72 h for 5-AMQ and both CDDP concentrations (Figure 8F,G), as well as for EL-1 and 10 nM CDDP (Figure 8H). In the MCC13 cell line, 5-AMQ induced an increase in chemosensitivity at all time points for 1 nM CDDP and 24 h for 10 nM CDDP (Figure 8I,J). On the contrary, the same effect was exerted by 6MeONa with 1 nM CDDP at 24 h (Figure 8K). Surprisingly, combined treatment significantly (*p* < 0.05) improved the effect induced by a single treatment in at least one time point for all combinations: 5-AMQ + 1 nM CDDP at 48 h (Figure 8I), 5-AMQ + 10 nM CDDP at all time points (Figure 8J), 6MeONa + 1 nM CDDP at 24 h and 48 h (Figure 8K) and 6MeONa + 10 nM CDDP at 24 h (Figure 8L). As for MCC26, 5-AMQ did not significantly exhibit a strong ability in potentiating the cytotoxic effect of CCDP regarding the explored combinations (Figure 8M,O), while EL-1 displayed its efficacy for both CCDP concentrations at 72 h (Figure N,P). As for the combined treatment, the improved efficacy with respect to a single administration was detectable with 5-AMQ + 10 nM CDDP at 48 h and 72 h (Figure 8O), EL-1 + 1 nM CDDP at 48 h (Figure 8N) and EL-1 + 10 nM CDDP at 48 h (Figure 8P).

### 3.7. Impact of Treatment with NNMT Inhibitors Combined with Chemotherapy on Intracellular Oxidative Stress

**Figure 9:**
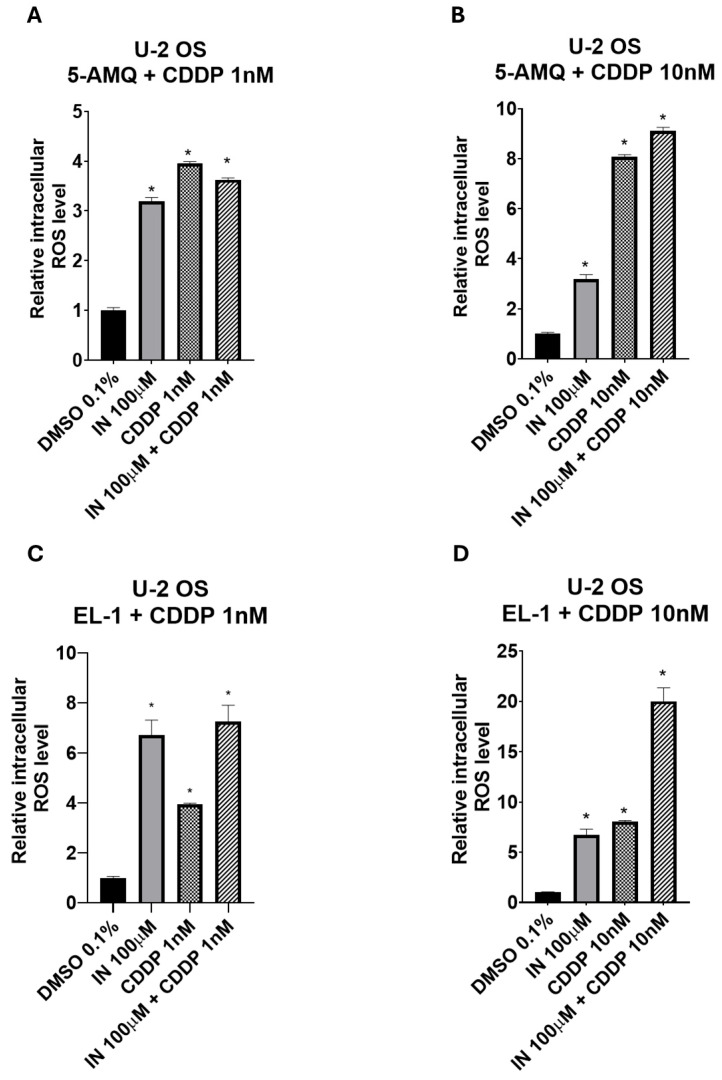

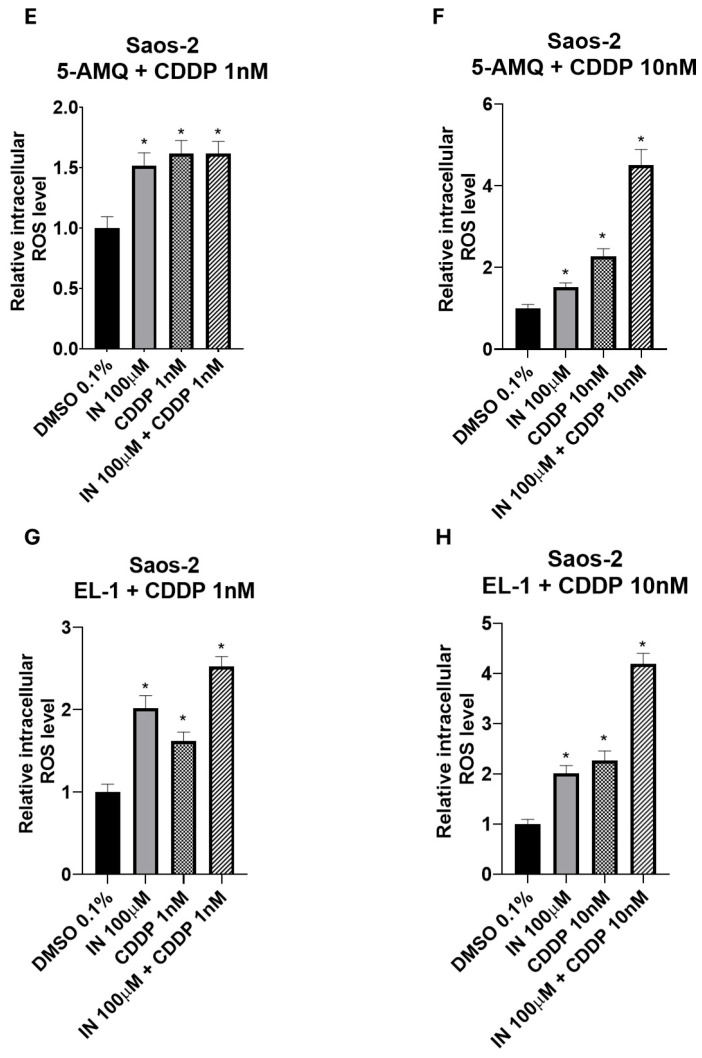

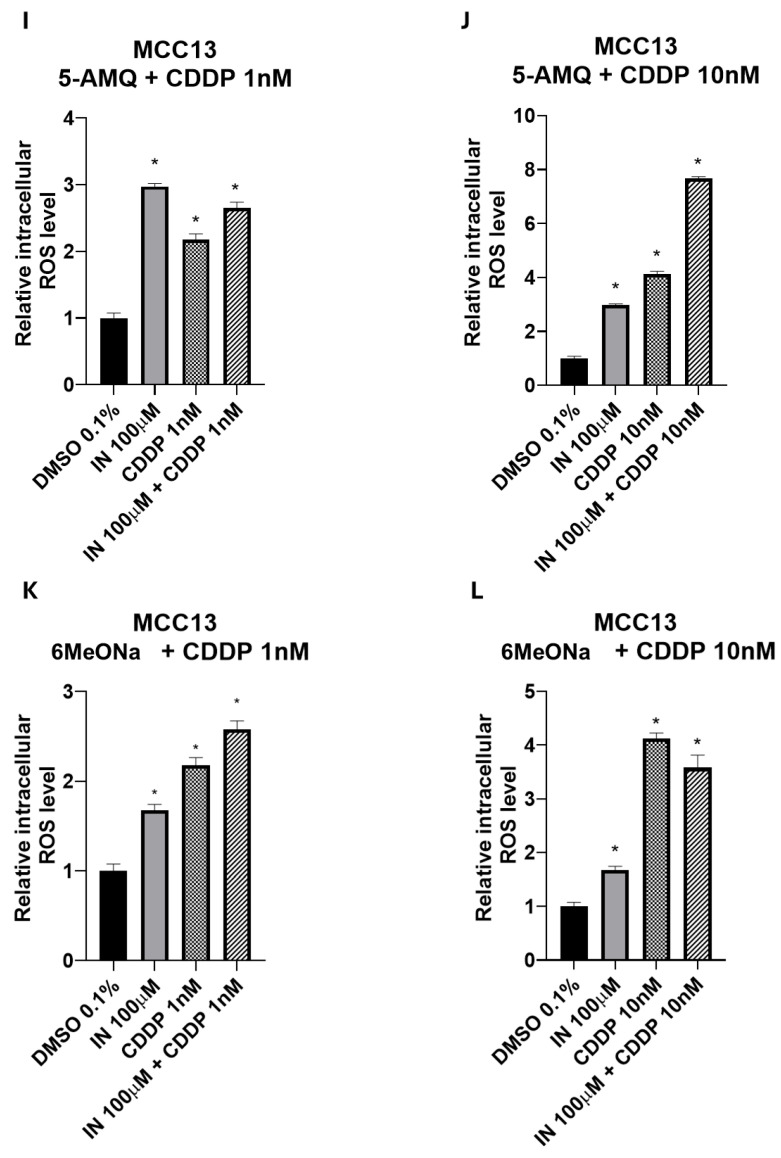

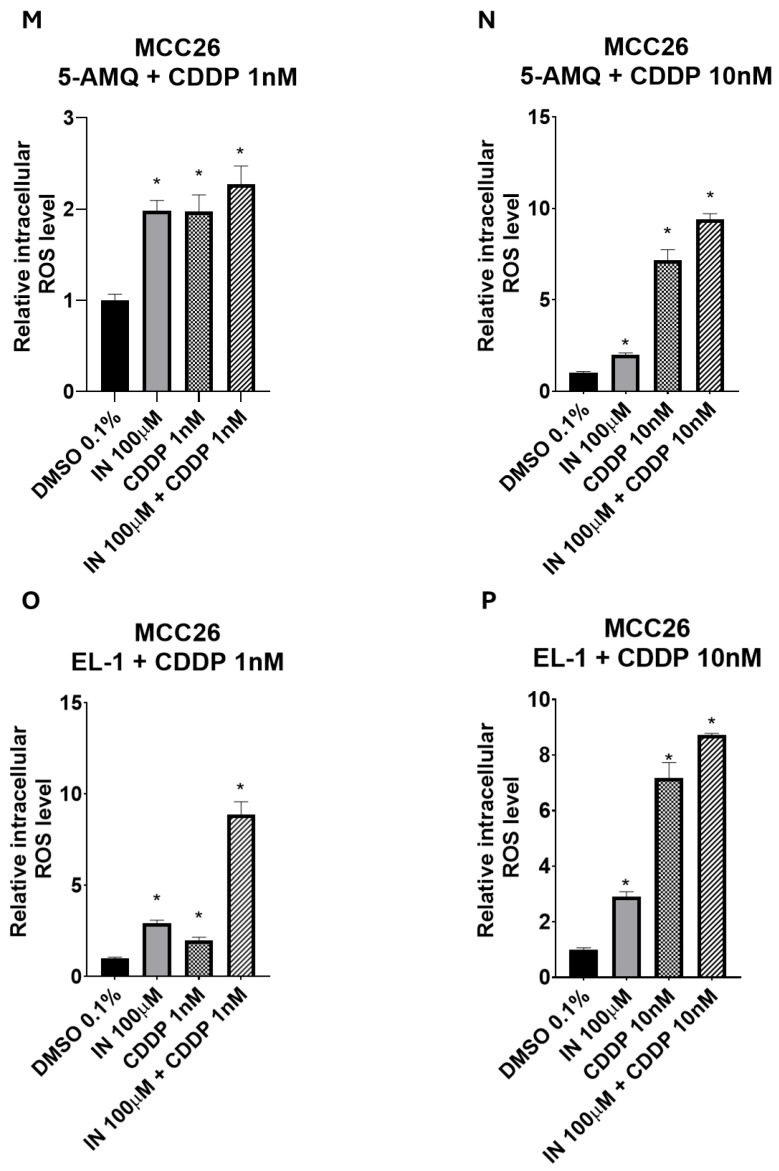
Combined effect of NNMT inhibitors and chemotherapy drug cisplatin (CDDP) on oxidative stress of OS and MCC cell lines. U-2 OS (**A**–**D**), Saos-2 (**E**–**H**), MCC13 (**I**–**L**) and MCC26 (**M**–**P**) cells were incubated with NNMT inhibitors 5-AMQ, 6-MeONa or EL-1 (100 μM) or CDDP (1 nM and 10 nM), used alone or in combination, while control cells were treated with DMSO (0.1%). ROS production was determined at 72 h after treatment by using DCFH2-DA probe. All values are expressed as mean ± standard deviation (* *p* < 0.05).

To investigate biochemical events potentially promoting tumor cell chemosensitivity, the impact of 100 µM NNMT inhibitor treatment in association with 1 or 10 nM CDDP was investigated in terms of intracellular oxidative stress. In the U-2 OS cell line, EL-1 was able to enhance the increase in ROS production with respect to that obtained following treatment with 1 nM CDDP only (Figure 9C). Interestingly, the combined treatment was significantly (*p* < 0.05) more efficient in amplifying ROS levels with respect to a single administration when EL-1 was used with 10 nM CDDP (Figure 9D). Similarly, also in Saos-2 cells, the treatment with EL-1 alone was slightly but significantly (*p* < 0.05) more efficient in enhancing ROS production than that detected under administration of 1 nM CDDP (Figure 9G). Both 5-AMQ and EL-1 used in combination with the highest CDDP concentration were able to increase oxidative stress at a higher extent than that observed under a single administration (Figure 9F,H). In MCC13, 5-AMQ administration led to a higher increase in ROS production compared with that induced upon treatment with 1 nM CDDP (Figure 9I). On the contrary, 6MeONa used alone did not exhibit any significant power in amplifying the effect generated by both CDDP concentrations (Figure 9K,L). Surprisingly, the combination of 6MeONa + 1 nM CDDP and even more 5-AMQ + 10 nM CDDP significantly (*p* < 0.05) enhanced the levels of ROS with respect to that observed under the treatments with single agents (Figure 9K,J). In MCC26, EL-1 appeared to be more efficient in raising oxidative stress than 1 nM CDDP (Figure 9O). Notably, in all combined treatments of both NNMT inhibitors and CDDP at different concentrations, ROS levels were significantly (*p* < 0.05) higher than those generated upon single treatments (Figure 9M–P), thus reaching the highest efficiency with EL-1 + 1 nM CDDP combination (Figure 9O).

## 4. Discussion

Current therapeutic options for localized OS treatment include surgical resection and systemic polychemotherapy (doxorubicin, methotrexate, cisplatin and ifosfamide), which are able to successfully treat ~70% of patients. However, patients with relapsed, locally advanced or metastatic disease display an overall 5-year survival rate of about 20% [29]. Clinically, OS is characterized by a high tendency to metastasize and frequent refractoriness to chemotherapy, thus limiting the effectiveness of cytotoxic drugs and contributing to poor prognosis in patients affected from this rare but aggressive neoplasm [30]. As for MCC, wide local excision, followed by lymph node dissection in the case of clinically node-positive disease, represents the standard of care for localized or loco-regional forms. Radiotherapy may be received by patients who refuse surgery or are not surgical candidates, it but can also be adopted to treat tumors that cannot be completely excised. Before the introduction of immunotherapy in 2016, metastatic MCC not amenable to surgery was treated with chemotherapy, resulting in complete or partial response rates in ~50% cases [31,32]. In the light of these considerations, the elucidation of the mechanisms of chemoresistance, together with the implementation of strategies to overcome it, are key to improving the survival rate of OS and MCC patients. To this end, the identification of molecular targets and new insights on their cellular functions have led to the development of small molecule inhibitors as major therapeutic compounds for cancer treatment, contributing to the progress of targeted therapies, also known as precision or personalized medicine [33].

In the present study, we focused our investigations on exploring the potential of three small molecules inhibitors of NNMT: 5-AMQ, 6MeONa and EL-1 [12]. Both 5-AMQ and 6MeONa represent inhibitors competing with the NA substrate for binding of the enzyme active site. NNMT activity in vitro assays, carried out using homogeneously purified human recombinant NNMT and with substrates combined with inhibitors, led to the construction of inhibition curves that were used to calculate 1.2 ± 0.1 μM [26] and 2.5 ± 0.1 μM [27] IC50 values for 5-AMQ and 6MeONa, respectively. In contrast, EL-1 represents a dual-substrate competitive inhibitor, able to interfere with NNMT catalysis by engaging both NA and SAM binding sites. In this regard, EL-1 is reported to belong to a novel class of pyrimidine-5-carboxamide compounds as inhibitors of NNMT, exhibiting a markedly higher inhibition potency (IC50 = 74 nM) [28].

Although the impact of these compounds on NNMT inhibition has been extensively investigated through in vitro biochemical assays, few data are available regarding their effects in cell-based assays. To address this issue, and building on our previously gained knowledge about the effect induced on tumor cell phenotypes by NNMT in association with OS and MCC, we here report the first investigations of the small-molecule enzyme inhibitors 5-AMQ, 6MeONa and EL-1 to modulate viability, apoptosis rate, ROS production and sensitivity to cisplatin treatment of two osteosarcoma (U-2 OS and Saos-2) and two Merkel cell carcinoma (MCC13 and MCC26) cell lines. The results obtained showed that cells treated with these NNMT inhibitors underwent significant reductions in metabolic activity and vitality, enhanced ROS production and activation of apoptotic pathways. Notably, compounds used to treat OS and MCC cells were able to increase their sensitivity to CDDP’s antiblastic effect, probably due to their capacity to increase the levels of oxidative stress mediators.

As reported by Neelakantan et al., who first investigated the properties of 5-AMQ as an anti-obesity drug, the compound displayed high permeability in terms of passive and active transport across membranes. Moreover, treating 3T3-L1 pre-adipocytes with 5-AMQ induced a significant decrease in cell viability in a dose-dependent manner and inhibited lipid accumulation (lipogenesis). 5-AMQ exhibited high selectivity, not inhibiting other SAM-dependent methyltransferases, like catechol-O-methyltransferase and nicotinamide phosphoribosyl transferase, or enzymes belonging to the NAD+ salvage pathway, such as nicotinamide phosphoribosyl transferase. The data obtained by in vivo analyses revealed that subcutaneous injection of 5-AMQ in diet-induced obese C57Bl/6 mice caused significant weight loss, reduced adipose tissue mass, improved oral glucose tolerance and insulin sensitivity, and suppressed hyperinsulinemia. In the liver, hepatic steatosis, macrophage infiltration and triglyceride levels, together with organ weight and size, were markedly reduced. Moreover, 5-AMQ treatment normalized circulating levels of alanine transaminase, aspartate transaminase and ketone bodies [34,35]. As for skeletal muscle turnover during aging, in vivo experiments carried out in C57Bl/6 mice showed that 5-AMQ treatment increased the number of replicating muscle stem cells (muSCs) that were promoted to fuse into large sized myofibers provided with a greater contractile force, thus suggesting improved muscle regeneration following injury. Further in vitro experiments, conducted in C2C12 myoblasts mirroring many traits of muSCs, demonstrated that treatment with 5-AMQ increased myoblast differentiation [36].

Analogously to 5-AMQ, in C57BL/6 mice with high-fat diet-induced obesity, 6MeONa treatment caused a reduction in body weight, improved insulin sensitivity and normalized glucose tolerance to the level of lean control mice [37]. In PC9 and HCC827 non-small cell lung cancer cell lines resistant to antiblastic treatment with a tyrosine kinase inhibitor targeting epidermal growth factor receptor and overexpressing NNMT, administration of 6MeONa suppressed growth both in vitro and in vivo [38]. The treatment of mouse xenografted tumors derived from human gallbladder carcinoma cells overexpressing NNMT with 6MeONa was associated with compromised tumor progression [39].

Regarding EL-1, the capacity of the small molecule to inhibit NNMT catalytic activity has been investigated in vitro by using biochemical inhibition assay with human recombinant NNMT [28]. However, no further functional studies were carried out, either in cell lines or in animal models, to evaluate the decrease in MNA levels associated with enzyme inhibition, as well as the potential effect of small molecule NNMT inhibitor treatment on phenotype or morphology.

Interestingly, enhanced sensitivity to treatment with CDDP, exhibited by OS and MCC cells exposed to different concentrations of small molecule NNMT inhibitors, was associated with increased ROS levels and apoptosis activation state. The reason for this association is not purely coincidental but rather the consequence of the mechanism of action of CDDP, on one side, and of the role played by NNMT within intracellular oxidative balance, on the other side.

Upon hydrolytic displacement of chloride atoms from the platinum-derived anti-neoplastic agent, previously internalized by active transport or passive diffusion, the remaining product becomes a potent electrophilic species able to efficiently react with nitrogen atoms of nucleic acid bases, thus increasing DNA damage [40]. Cisplatin also induces oxidative stress by increasing ROS release and reducing the oxidant scavenging capacity that triggers cell death in addition to DNA damage. Excessive reactive oxygen species can induce apoptosis through both the extrinsic and intrinsic pathways, respectively initiated by caspase-8 and caspase-9, but further commonly propagated by the executioner caspase-3, -6 and -7 [41]. Based on what is reported in the literature, enzyme dysregulation in cancer cells was significantly correlated to impairment of mechanisms featuring oxidative stress. The induction of NNMT expression in the human neuroblastoma cell line SH-SY5Y led to a significant increase in total glutathione (GSH), free GSH and its oxidized form (GSSG), together with a reduction in the GSH:GSSG ratio, as well as in ROS content. Taken together, these data seem to suggest a potential role of the enzyme in reducing ROS-related oxidative stress by enhancing the buffering capacity of GSH [42]. NNMT upregulation in the SK-BR-3 breast cancer (BC) cell line, lacking endogenous NNMT expression, greatly reduced ROS-associated autophagy, while this effect was reversed following enzyme knockdown in MDA-MB-321 BC cells, originally displaying elevated NNMT levels. These data strongly indicate the potential capacity of NNMT to exert a protective effect on breast cancer cells, protecting against oxidative stress through autophagy suppression [43]. In light of these considerations, it is conceivable to expect that reductions in cell viability following combined treatment with CDDP and NNMT inhibitors appear additionally or synergistically higher than those observed in cells separately treated with single agents.

## 5. Conclusions

In conclusion, the findings here reported demonstrated that cells treated with NNMT inhibitors undergo significant reductions in metabolic activity and viability, enhanced ROS production and activation of apoptotic pathways. Moreover, compounds used to treat OS and MCC cells were able to increase cell sensitivity to the cytotoxic effect of CDDP. Further analyses will be carried out to clarify the molecular mechanisms responsible for the aggressive tightening of the tumor cell phenotype, where NNMT participation is believed to be involved.

A limitation of the study could be represented by the fact that neither the selectivity of small molecule NNMT inhibitors nor their potential off-target effects were explored. Indeed, most drug-like compounds modulate their target molecules, leading either to therapeutic effects or unwanted side effects, with such target promiscuity being partly responsible for the high attrition rates and wasted costs and time in the drug discovery process [44].

However, due to the pivotal role played by NNMT in the deleterious behavior of cancer cells, targeting NNMT might represent an interesting strategy for counteracting tumor growth and chemoresistance, thus providing a rationale for novel anti-cancer therapies based on the inhibition of NNMT.

## Data Availability

The data presented in this study are available on request from the corresponding author. The data are not publicly available due to privacy.

## Supplementary Materials

The following supporting information can be downloaded at: https://www.mdpi.com/article/10.3390/biom15111553/s1, Original data from WB reported to in Figure 2.

## Abbreviations

The following abbreviations are used in this manuscript:

BC	Breast Cancer
CDDP	Cisplatin
DCFH2-DA	2′,7′-Dichlorodihydrofluorescein Diacetate
DMSO	Dimethyl Sulfoxide
EL-1	Eli Lilly’s Pyrimidine 5-Carboxamide
5-AMQ	5-Amino-1-Methyl Quinolinium
GSH	Reduced Glutathione
GSSG	Oxidized Glutathione
MCC	Merkel Cell Carcinoma
MNA	N1-Methylnicotinamide
MTT	(4,5-dimethylthiazol-2-yl)-2,5-diphenyl tetrazolium bromide
muSCs	Muscle Stem Cells
NA	Nicotinamide
NAD^+^	Oxidized Nicotinamide Adenine Dinucleotide
NNMT	Nicotinamide N-Methyltransferase
OS	Osteosarcoma
ROS	Reactive Oxygen Species
SAH	S-Adenosyl-L-Homocysteine
SAM	S-Adenosyl-L-Methionine
6MeONa	6-Methoxynicotinamide
