# Supplementary material for: Small Molecule Inhibitors of Nicotinamide N-Methyltransferase Enzyme for the Treatment of Osteosarcoma and Merkel Cell Carcinoma: Potential for the Development of a Targeted Therapeutic Strategy
Source: Biomolecules. 2025 Nov 5;15(11):1553. doi: 10.3390/biom15111553 (PMC12650368; doi:10.3390/biom15111553)
Supplement: Supplementary file 1 [file biomolecules-15-01553-s001.zip › biomolecules-3780907-blot images/BLOTS.pdf]

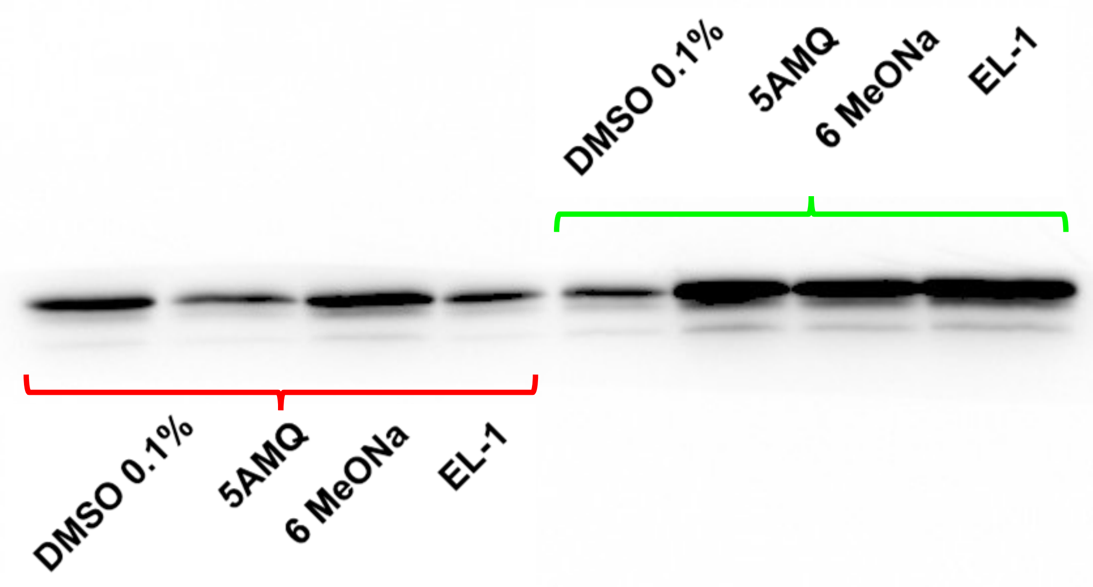

Caspase-3 bands in U2OS and Saos-2 cells

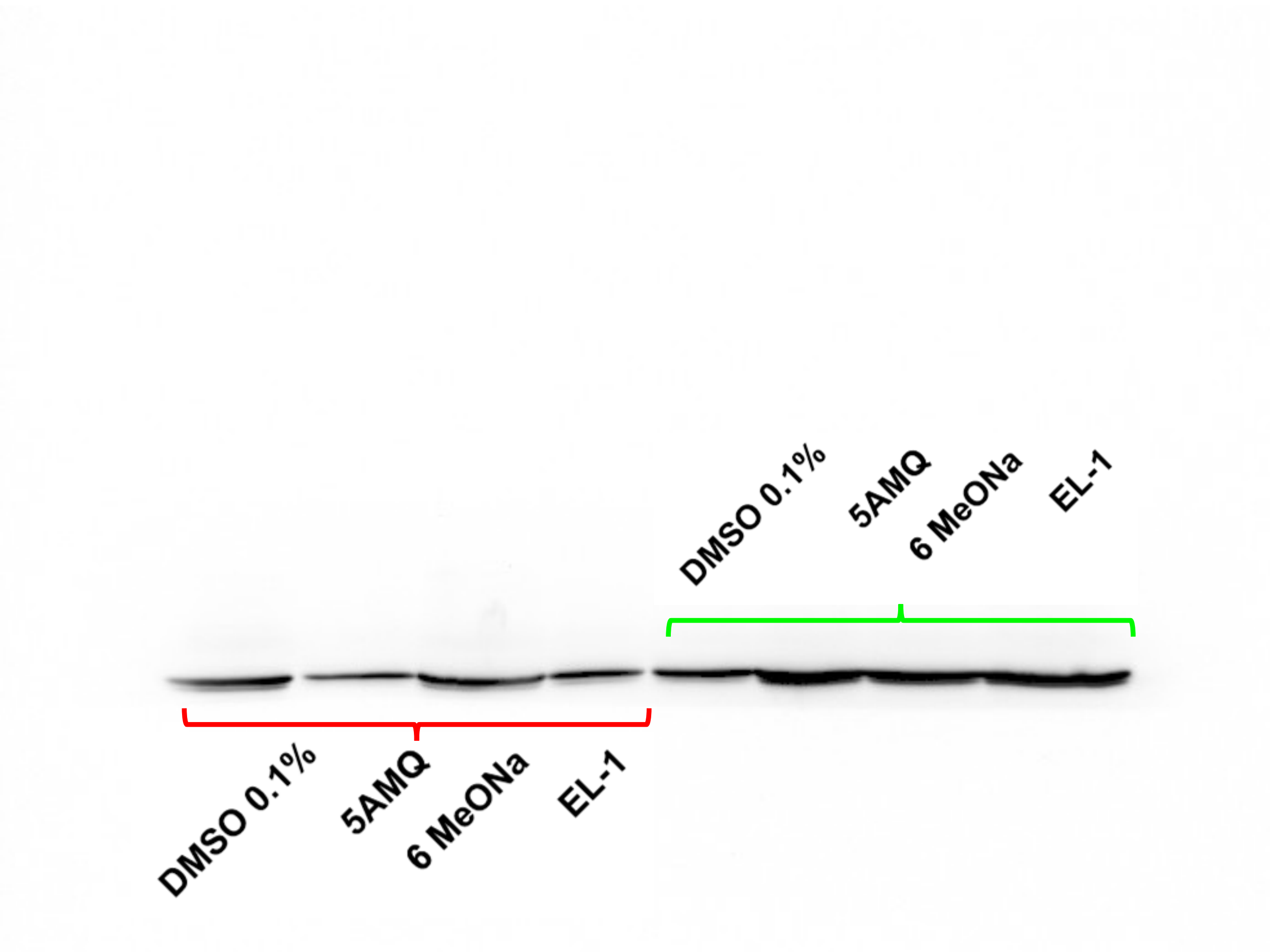

β-actin bands in U2OS and Saos-2 cells

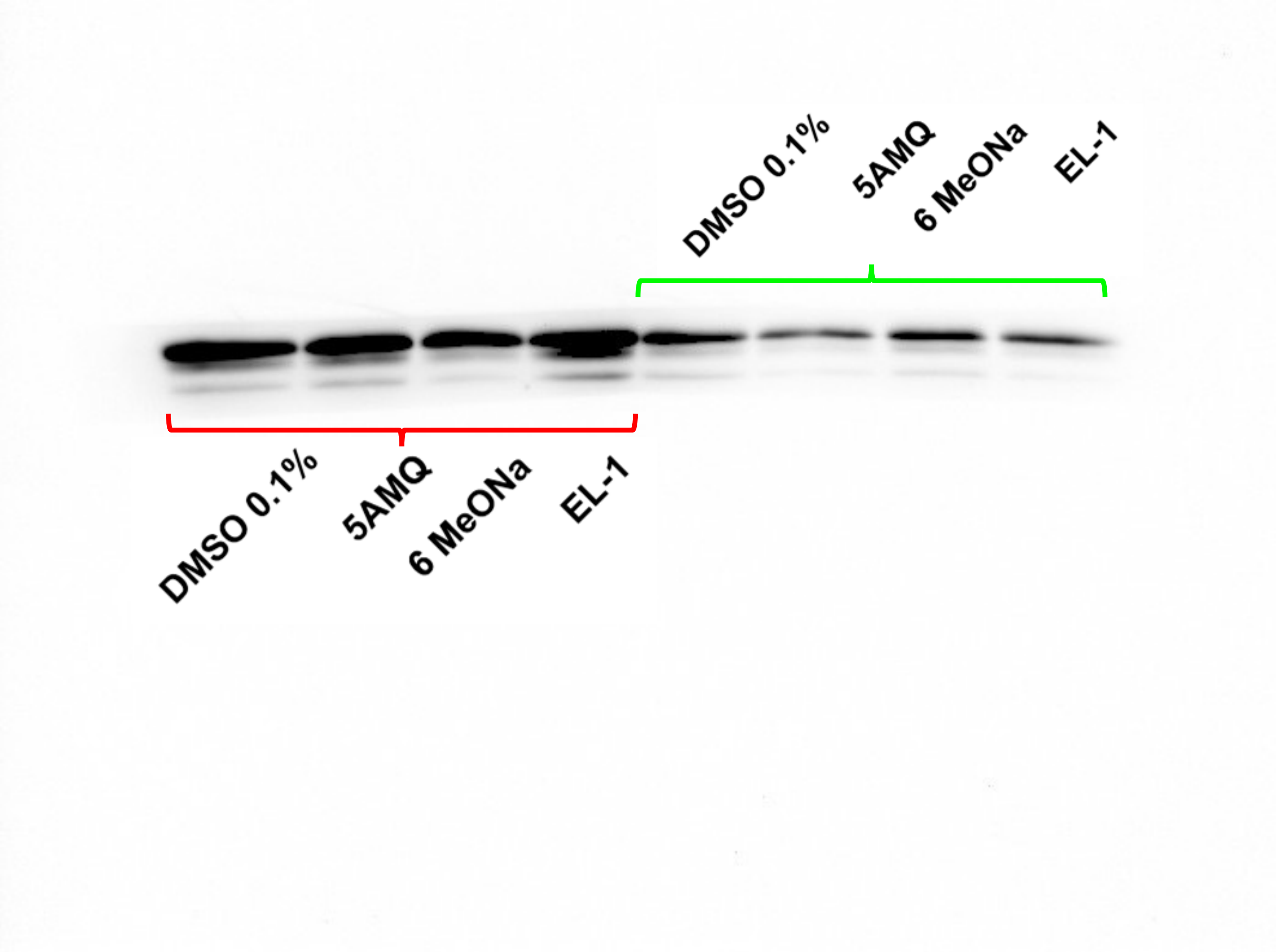

Caspase-3 bands in MCC13 and MCC26 cells

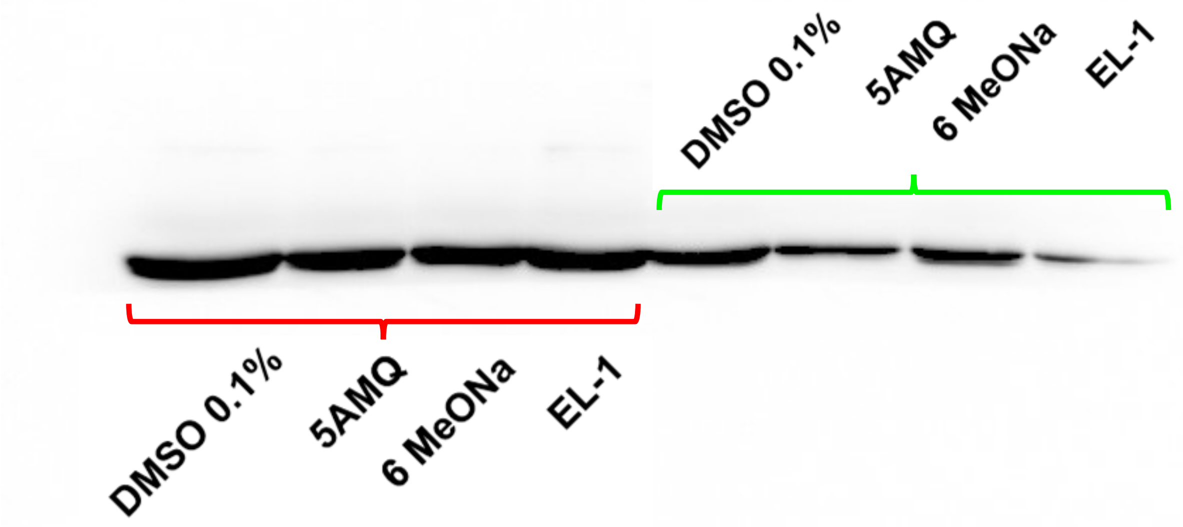

β-actin bands in MCC13 and MCC26 cells
